# A Rare Complication of Supraglottic Myxedema Secondary to Hypopituitarism and Hypothyroidism

**DOI:** 10.7759/cureus.81374

**Published:** 2025-03-28

**Authors:** Retaj Alawadhi, Sood Alsairefi, Imtiyaz N Bhat, Javeed I Kachroo, Mishal M AlMutairi

**Affiliations:** 1 Medicine and Surgery, Royal College of Surgeons in Ireland, Dublin, IRL; 2 Otolaryngology-Head and Neck Surgery, Farwaniya Hospital, Al Farwaniyah, KWT; 3 Otolaryngology–Head and Neck Surgery, Farwaniya Hospital, Al Farwaniyah, KWT

**Keywords:** acute follicular tonsillitis, hypothyroidism, pituitary adenoma, supraglottic edema, supraglottic myxedema

## Abstract

Supraglottic edemas are rare complications of severe hypothyroidism that present significant challenges in management due to their critical location and acute clinical manifestations. The traditional approach of managing hypothyroidism and airway support has been the preferred treatment in the case of supraglottic edema, while glucocorticoids have a controversial therapeutic benefit. Here, we present a case of a 59-year-old male patient with a history of hypothyroidism and pituitary adenoma resection who presented to the emergency department with stridor and shortness of breath, where he underwent ICU admission, was sedated, and received medical treatments. The pre-ICU fiberoptic evaluation suggested supraglottic edema and congestion of the nasopharynx, and neck imaging revealed an inflammatory process of the right side of the oropharynx and right palatine tonsil and ruled out the presence of an abscess in deep neck spaces. Intensive care unit stay and the multidisciplinary treatments were beneficial, resulting in complete remission of the supraglottic edema and the subsequent symptoms. This case highlights the manifestation of supraglottic edema as a complication of hypothyroidism post pituitary adenoma resection and emphasizes the importance of multidisciplinary collaboration in achieving successful outcomes of these challenging presentations.

## Introduction

Supraglottic edema is a rare but potentially life-threatening complication often associated with severe hypothyroidism. This condition presents significant challenges in clinical management due to its critical location and acute manifestations, such as airway obstruction. The traditional approach to managing supraglottic oedema involves addressing the underlying hypothyroidism and providing airway support. However, the use of glucocorticoids remains controversial [1,2]. Here, we present a case of a 59-year-old male patient with a history of hypothyroidism and pituitary adenoma resection, who presented with stridor and shortness of breath. The use of glucocorticoids for supraglottic oedema as an adjust to the traditional approach of managing hypothyroidism is relatively controversial, and there is limited literature on its role. This case underscores the importance of a multidisciplinary approach in managing such complex presentations and highlights the potential for complete remission with appropriate treatment. 

## Case presentation

A 59-year-old male patient with a known history of hypothyroidism and previous pituitary adenoma resection presented to the emergency department with acute onset of stridor, shortness of breath, tachypnea, and dysarthria. The patient reported experiencing hoarseness of voice and difficulty swallowing for the past two days. On the day of admission, his symptoms worsened, prompting him to seek medical attention. He also reported a history of cigarette smoking, having quit one year prior, but otherwise had been in good health.

The patient, originally from Pakistan, faced a language barrier, which initially hindered the medical team from understanding his full medical history, including his known hypothyroidism. An interpreter was not used due to the unavailability at the time of admission, which delayed the full understanding of the patient's medical history. According to the patient, he had his pituitary adenoma resected in Pakistan in 2016 and has been on hydrocortisone 100 mg and levothyroxine 100 mcg tablets since his operation, with recurrent attacks of tonsillitis/sore throat. The patient was compliant with his medication regimen, as he had been taking hydrocortisone and levothyroxine regularly since his surgery. Upon his current hospital presentation, the patient was evaluated by ENT and underwent ICU admission, where he was sedated and received multiple medical treatments, including antibiotics and steroids.

**Figure 1:**
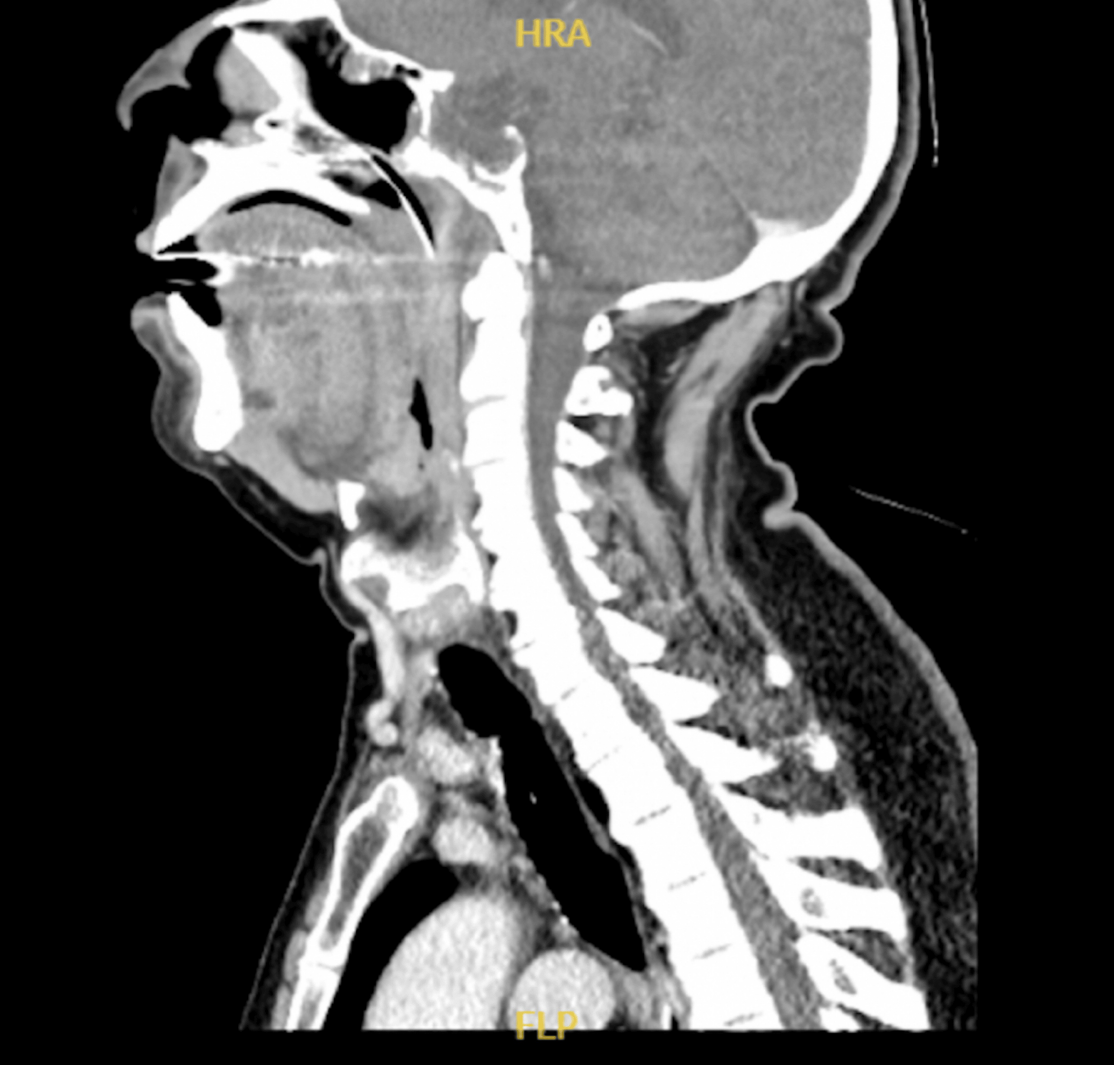
Sagittal ceck CT with IV contrast Inflammatory process of the right side of the oropharynx and right palatine tonsil, with no evidence of localized fluid collections or abscesses

The pre-ICU admission assessment involved ENT and neck imaging evaluations. The ENT evaluation by fiberoptic exam confirmed uvular, supraglottic edema, and nasopharyngeal congestion in addition to the presence of acute follicular tonsillitis. A CT scan of the neck with contrast was requested to rule out any abscess formation in deep neck spaces. Results revealed an inflammatory process of the right side of the oropharynx and right palatine tonsil, with no evidence of localized fluid collections or abscesses (Figure 1). Scanned lung apices showed bilateral apical atelectatic bands and pleuro-pulmonary reaction.

Given the severity of his symptoms and the risk of airway obstruction, the patient was admitted to the ICU for close monitoring and management. He was sedated and intubated to secure the airway, and he was started on intravenous hydrocortisone, omeprazole, and broad-spectrum antibiotics. As previously mentioned, due to the language barrier, the patient was not found to have hypothyroidism until later on during his hospital stay. Thus, the initial diagnosis was acute follicular tonsillitis with supraglottic edema; a multidisciplinary approach was initiated to manage his complex condition.

During his ICU stay, the patient was assessed and followed by ENT, internal medicine, and endocrinology. Based on ENT daily evaluations, which showed persistent edematous epiglottitis, right-sided pharyngeal wall edema, and vocal cords not being visualized due to thick secretions, the decision was made to keep the patient under sedation until the airway was patent. The patient continued to receive hydrocortisone 100 mg four times a day (QID), clindamycin, ceftriaxone, and enoxaparin. Internal medicine recommended resuming levothyroxine 75 mcg and consulting endocrinology for further assessment. Endocrinology evaluation confirmed decreased levels of morning cortisol (45 nmol/l) and recommended starting hydrocortisone 50 mg every eight hours, in addition to tracing thyroid function tests (TFTs), follicle-stimulating hormone (FSH), prolactin, and total testosterone levels.

Over the subsequent days, the patient’s condition gradually improved. On the third day, his fiber-optic exam done by the ENT team showed remission of supraglottic edema with the presence of thick secretions only. The patient was successfully extubated on the fifth day and transitioned to high-flow nasal cannula (HFNC) for oxygen support. His condition continued to improve, and he was later transitioned to room air. Throughout his ICU stay, the patient was monitored closely, with adjustments to his treatment plan based on clinical progress and laboratory results. He was eventually discharged to the ward, maintaining stable vital signs and oxygen saturation.

Follow-up care included continued levothyroxine and hydrocortisone therapy, with scheduled evaluations by ENT and endocrinology specialists to monitor for any recurrence of edema or hormonal imbalances. The patient showed smooth recovery without complications and was discharged after a five-day hospital stay. This case emphasizes the crucial role of a multidisciplinary approach in facilitating a patient’s recovery and highlights the importance of coordinated care in managing complex cases of supraglottic edema secondary to hypothyroidism and post-acute follicular tonsillitis.

## Discussion

Supraglottic edema is a rare but serious complication of hypothyroidism, particularly in patients with a history of pituitary adenoma resection. Other causes of supraglottic edema can include infections, allergic reactions, trauma, and systemic diseases such as amyloidosis. The management of such cases requires a multidisciplinary approach involving ENT specialists, intensivists, and endocrinologists. Our case shares similarities with the two cases reported in the literature by Del Prado et al [1].

In the first case reported by Del Prado et al., a 63-year-old man with a history of obesity, sleep apnea, and hypertension presented with confusion, somnolence, hoarseness, and dyspnea. He was noted to have facial and neck edema and was subsequently intubated for airway protection. Thyroid function testing revealed elevated thyroid-stimulating hormone (TSH) and low free thyroxine (FT4), consistent with severe hypothyroidism. The patient was treated with intravenous hydrocortisone and levothyroxine, resulting in symptomatic improvement and resolution of airway edema [1].

The second case involved a 70-year-old male patient with a history of papillary thyroid cancer treated with total thyroidectomy and radioactive iodine. He presented with a sore throat, stridor, and lower eyelid edema. Fiberoptic examination revealed supraglottic edema, and CT imaging showed prominent pharyngeal mucosal thickening with retropharyngeal edema. The patient was managed with intravenous levothyroxine and dexamethasone, leading to significant improvement [1].

Our patient, a 59-year-old male patient with a history of hypothyroidism and pituitary adenoma resection, presented with similar symptoms of hoarseness, shortness of breath, and stridor. The initial management included corticosteroids, antibiotics, and supportive care. The patient required intubation and ICU admission due to respiratory distress. Fiberoptic examination confirmed supraglottic edema, and neck imaging revealed an inflammatory process in the oropharynx and palatine tonsil and ruled out abscess in deep neck spaces. The multidisciplinary approach, which involved ENT, internal medicine, and endocrinology consultations, was crucial in managing the patient’s condition. The patient showed significant improvement and was extubated after five days, similar to the cases reported in the literature.

The use of corticosteroids in managing supraglottic edema remains controversial. While some studies suggest that corticosteroids can reduce inflammation and edema, others argue that their benefits are not well-established [1,2]. In our case, the administration of high-dose corticosteroids, along with thyroid hormone replacement and antibiotics, was effective in reducing the edema and resolving the patient’s symptoms. This aligns with the positive outcomes observed in the literature.

Several studies have highlighted the mechanisms of edema formation in myxedema, including increased protein extravasation and relatively slow lymphatic drainage. The identification of thyroid hormone receptors in the human larynx further supports the role of thyroid hormone replacement in managing laryngeal myxedema [3]. Additionally, the management of hypothyroidism is crucial in the context of supraglottic edema threatening airway patency in an ICU setting. Failure of careful monitoring and adjustment of thyroid hormone levels could potentially lead to serious complications and ultimately death [4-6].

The rarity of supraglottic edema as a complication of hypothyroidism, particularly in the context of post-pituitary adenoma resection, adds to the complexity of managing such cases. The literature suggests that early intervention with corticosteroids and thyroid hormone replacement can lead to significant improvement in symptoms and prevent airway obstruction [1,5].

In our case, the patient’s history of pituitary adenoma resection and subsequent hypothyroidism likely contributed to the development of supraglottic edema. The multidisciplinary approach, involving ENT, endocrinology, and critical care specialists, was essential in managing the patient’s condition effectively. The patient’s positive response to corticosteroids and thyroid hormone replacement underscores the importance of addressing the underlying endocrine disorder in managing airway edema. As with any critical patient, a multidisciplinary team and careful patient factors are essential to ensure ideal outcomes.

## Conclusions

The presented case highlights the successful application of early intervention and multidisciplinary treatments in managing supraglottic edema and acute follicular tonsillitis as a manifestation of hypothyroidism post-pituitary adenoma resection. The patient was admitted to the hospital for a total of five days, during which he received comprehensive care. Supraglottic edemas, particularly in the case of hypothyroidism, are rare and present unique challenges. The use of corticosteroids in managing supraglottic edema remains controversial. However, in this case, the use of corticosteroids in addition to a broad spectrum of antibiotics allowed for complete remission of supraglottic edema and the rest of the subsequent symptoms. Factors such as past medical history of hypothyroidism, hormonal imbalance, and the cause behind it should guide the patient management. This case report contributes to the limited literature on supraglottic edema in the context of hypothyroidism post pituitary adenoma resection. It emphasizes the importance of a multidisciplinary approach and the need for ongoing research on the role of steroids in such cases to optimize outcomes for patients with these critical presentations.
